# Long-Term Impact of Wind Erosion on the Particle Size Distribution of Soils in the Eastern Part of the European Union

**DOI:** 10.3390/e23080935

**Published:** 2021-07-22

**Authors:** Lenka Lackóová, Jozefína Pokrývková, Jana Kozlovsky Dufková, Agnieszka Policht-Latawiec, Krystyna Michałowska, Jolanta Dąbrowska

**Affiliations:** 1 Department of Landscape Planning and Ground Consolidation, Faculty of Horticulture and Landscape Engineering, Slovak University of Agriculture in Nitra, 949 76 Nitra, Slovakia; lenka.lackoova@uniag.sk; 2 Department of Water Resources and Environmental Engineering, Faculty of Horticulture and Landscape Engineering, Slovak University of Agriculture in Nitra, 949 76 Nitra, Slovakia; jozefina.pokryvkova@uniag.sk; 3 Department of Applied and Landscape Ecology, Faculty of AgriSciences, Mendel University in Brno, 613 00 Brno, Czech Republic; jana.dufkova@mendelu.cz; 4 Department of Land Reclamation and Environmental Development, Faculty of Environmental Engineering and Land Surveying, University of Agriculture in Krakow, 30-059 Kraków, Poland; a.policht@urk.edu.pl; 5 Department of Geodesy, Faculty of Civil and Environmental Engineering, Gdańsk University of Technology, 80-233 Gdańsk, Poland; 6 Department of Geodesy, Faculty of Environmental Engineering and Land Surveying, University of Agriculture in Krakow, 30-059 Kraków, Poland; 7 Department of Civil Engineering, Faculty of Environmental Engineering and Geodesy, Wrocław University of Environmental and Life Sciences, 50-363 Wrocław, Poland; jolanta.dabrowska@upwr.edu.pl

**Keywords:** aeolian erosion, land degradation, GIS, sustainable agroecosystems, decision-making processes, soil protection, wind erosion indicators, landscape entropy and stability

## Abstract

Wind erosion is the leading cause of soil degradation and air pollution in many regions of the world. As wind erosion is controlled by climatic factors, research on this phenomenon is urgently needed in soil and land management in order to better adapt to climate change. In this paper, the impact of wind erosion on the soil surface in relation to particle size distribution was investigated. Changes in percentage of sand, silt and clay fractions based on historical KPP data (1961–1970), LUCAS data base (2009), and field measurements (2016) were analysed in five cadastral areas impacted by wind erosion (Záhorie Lowlands, Slovakia). With the use of GIS tools, models of spatial distribution of sand, silt, clay and erodible fraction (EF) content were developed based on those measurements. Our findings proved that soil texture change driven by wind erosion could happen relatively quickly, and a significant proportion of soil fine particles may be carried away within a few years. The results indicate that the soil surface became much rougher over the period of more than 50 years, but also that the accumulation of fraction of the silt particles occurred in most of the areas affected by the erosive effect.

## 1. Introduction

Soil erosion is a major cause of global-scale land degradation, and has increased by 2.5% between 2001 and 2012. The main reasons for this increase are considered to be deforestation and cropland expansion [1,2]. Soil erosion by wind is the process of destruction, separation, transportation and deposition of soil particles that affects negatively land and human health, agricultural production, as well as ecosystem services [3,4,5]. The process is controlled by several factors such as: wind speed and direction, temperature, precipitation, soil wetness, surface roughness, soil texture and aggregation, soil organic matter, vegetation cover, field size, agricultural activities and protective measures [6,7]. Wind erosion involves three distinct steps: (1) Initiation of the soil particle movement (detachment and deflation); (2) Soil particle transportation (suspension, saltation and surface creep); (3) Deposition of soil particles [6].

The physical properties of the soil play an important role in determining the suitability of the soil for agricultural, environmental and constructional use [6,8,9]. They are directly linked not only to different soil functions, but also to processes in the soil, such as water transport, retention and availability of water, availability of plant nutrients, the ease of rooting, and the flow of heat and air [10,11,12,13,14,15]. Physical properties of the soil may be significantly affected in the long term by ongoing degradation processes. Wind erosion is a very effective sorting process. The coarsest particles remain on the soil, while the finer and most valuable soil particles like silt and clay [16], as well as the organic matter [17], are carried away by the wind. Urban et al. [18], by using the method of calculating the volume of eroded particles, found out that accumulation of eroded particles also plays an important role in wind erosion research. The change of soil surface affects the cycling of elements and energy in the environment, as well as the entropy and ecological stability of the landscape [19,20]. The process of removing particles by the wind is currently a problem of large landscape units [21]. Wind erosion quantification is based on the measurement of horizontal soil removal, which can be used to derive the soil loss/silt emissions of those particles or the deposition of transported particles [22,23].

Wind erosion is not only a problem that affects dryland regions, it happens, among others, also in the humid climate of northwestern Europe. Due to wind erosion, soil productivity is reduced for numerous arable lands in the EU. The loss of topsoil is the most significant effect of wind erosion in this part of the world [24,25,26,27,28]. Recently, Borrelli et al. [27] pointed out the need for a new phase of field measurements and local monitoring to increase the reliability of wind erosion modelling. This is necessary in order to meet the objectives of the EU Thematic Strategy for Soil Protection. Detailed quantitative studies examining the nutritional and chemical properties of sediments are lacking, and only a few are focused on the quantitative analysis of changes in particle size of the soil and eroded material caused by wind erosion. The scarcity of research may be surprising in view of the fact that about one third of the land surface area of the Earth has been affected by wind erosion [6,29,30]. The confluence of silt emissions influences physical and chemical processes in the atmosphere, as well as other ecosystems far from the source areas [31]. Dust and its chemical and biological components have a negative impact on air quality and thus pose a risk to human health [32,33,34]. To solve practical problems, for example of the channel flow, it is necessary to develop a method for calculating the distribution of average velocity of the suspended flow, which is used in wide variation ranges of the flow motion parameters conditions of the transported fluid and solid particles [35,36]. Cultivation of agricultural soils can also make a significant contribution to concentrations of particulate matter of 10 µm (PM_10_) in the air [37,38], which affects the quality of the environment and human health.

In many studies based on field measurements in the dry season [39,40,41], the percentage of particles <0.84 mm (obtained by sieving) was considered to be an indicator of soil erodibility by wind. This methodology has been used since the first prediction models [42,43]. Fryrear et al. [23] developed a multiple regression equation to calculate the erodible fraction of soils based on particle size and chemical properties in the soil [44]. Different types of soils have various erodibility due to differences in their structure, physical and chemical properties [45]. The basic concept of wind erosion is the function of soil erodibility and the erosive effect of wind.

Wind erosion physically removes the most fertile part of the soil surface, which leads to a decrease in soil nutrient content, thus not benefiting plant growth. Modelling spatial changes in the physical properties of soil is therefore essential, especially in areas where these phenomena occur frequently, so that we can understand their impacts and identify areas of conservation [3,46,47]. Many authors currently emphasise that research is particularly needed to support decision-oriented sustainable management frameworks for wind erosion prevention and control, encompassing erosion monitoring, assessment and management decisions [3,6,47,48,49]. Research related to soil and land management and environmental decision support systems is nowadays facilitated by increasing availability of high-resolution spatial data, remote sensing data and GIS analysis tools. Progressive climate change makes such studies a pressing need [50,51,52,53,54,55]. Current wind erosion models provide detailed knowledge necessary to predict and monitor this phenomenon and to implement effective mitigation measures, and are developed at different temporal and spatial scales using data integration from multiple sources [4,6,56,57]. The use of integrated information removes sources of uncertainty that disrupt wind erosion assessment and management [3].

Few studies [58,59,60,61] have yet been undertaken which have linked the quantitative effects of wind erosion to a detailed analysis of particle size fraction changes in the wind-damaged soil layer. This study examines the effect of wind erosion on the soil surface in relation to the size distribution of particles (changes in percentage of sand, clay and clay fractions) in the period of over 50 years (1961–2016). The main part of the research consists in analysing the impact of wind erosion on the change of particle size of the soil layer (<5 cm). The criteria for the selection of the study site were terrains with sandy soils susceptible to wind erosion in the Záhorie Lowlands in Slovakia.

The aim of the study is twofold: (i) to develop models of spatial distribution of sand, silt and clay content based on long-term archival data and current field measurements for selected cadastral units in the area where erosion processes occur, (ii) to investigate the degree of impact of erosion on changes in soil granulometric composition, which is important in soil management decision-making process.

## 2. Materials and Methods

### 2.1. Materials

**Figure 1:**
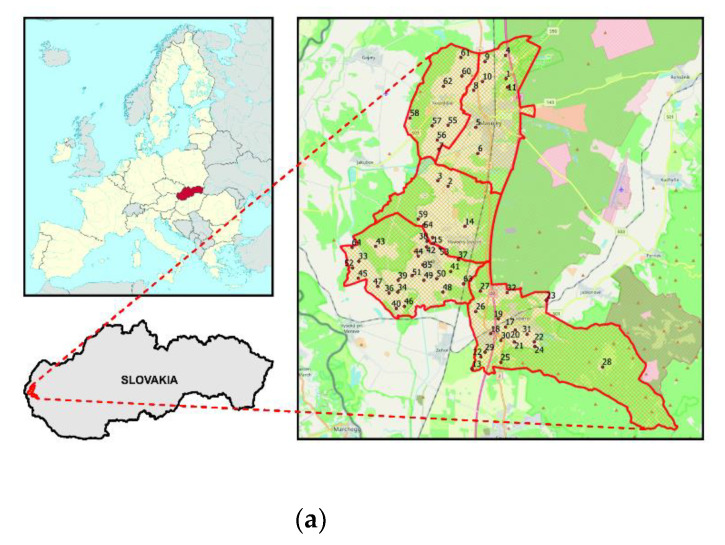

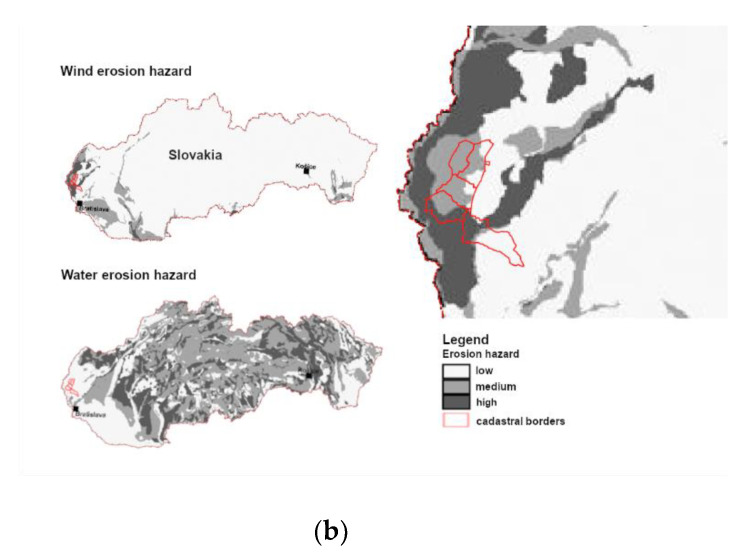
(**a**) The study area located in the eastern part of the European Union in the Záhorie Lowlands in Slovakia. (**b**) The study area on the map of wind and water erosion hazard in Slovakia—own elaboration based on the results of Minár et al. [62].

The research was carried out in the eastern part of the European Union in the Záhorie Lowlands. The research used data from field measurements made in five selected cadastral units: Kostolište, Láb, Lozorno, Malacky and Plavecký Štvrtok in the western part of Slovakia, with a total area of 14,074 ha. Soil samples were taken in spring, at the turn of April/May 2016. The research was conducted on the basis of 64 soil samples. The location of the samples was the same for the comprehensive soil assessment (KPP) from 1961–1970 and current field measurements in 2016. The research area with marked location of soil sampling points is shown in Figure 1a. The study site is located in an area of high and medium hazard of wind erosion, and there is no risk of water erosion (Figure 1b).

The KPP data set used in the research contains historical data from the national database of agricultural soils of the Slovak area from 1961–1970. The database collects data in electronic form on the percentage of soil particles: sand, silt and clay, and is made available by the Soil Research Institute (VÚPOP) in Bratislava.

In addition, the Land Use/Land Cover Area Frame Survey sampling of topsoil Land Survey (LUCAS 2009) was used together with the Topsoil Soil Map database [63]. The database is freely available (upon request) at the Joint Research Center website: esdac.jrc.ec.europa.eu/content/lucas-2009-topsoil-data (access on 05 January 2020). The data are made available in Excel format (LUCAS_TOPSOIL_v1).

The selection was made on the basis of preliminary analysis of land cover maps (LUCAS data base) and information on the size of particles in the soil in a given area.

### 2.2. Methods

In order to study changes in soil granulometric composition in the period of more than 50 years (1961 to 2016), data on the historical (KPP, LUCAS) and current state (2016) of soil particles were analysed. A total of 64 samples were selected in the cadastral areas of interest. Based on these data, the value of the proportion of sand, silt, and clay was identified for each sample using zonal statistics (ArcToolbox—Spatial Analyst Tools—Zonal—Zonal Statistics as Table) in ArcMap program 10.2.2. (Esri, Redlands, CA, USA). Zonal statistics calculates raster values within an identified zone of another database (e.g., points, polygons).

Comprehensive soil texture research in the years 1961–1970 (KPP) and samples from the European LUCAS system (2009) [63] were carried out by pipette method. Particle size analysis of current samples (2016) was determined using the ANALYSETTE 22 MicroTec plus laser analyser (FRITSCH GmbH, Idar-Oberstein, Germany). The particle sizes of fractions obtained with the pipette method cannot be directly compared to the size fraction determined by laser method. In order to standardize data, most researchers use regression analysis [64,65,66,67,68,69,70,71,72]. Igaz et al. [73] indicate the differences between the measured values by the pipette method and the Analysette22 MicroTec plus laser analyzer for the particle size fraction of <0.01 mm, in average from 3% up to 9% and after correction 3.28%. High correlation between the results of both methods was also confirmed by Kun et al. [74]. Balkovic et al. [75] created a spatial model (16,264 georeferenced samples) from sand and clay content in soils in the A horizon on the basis of samples from a comprehensive survey of agricultural soils. The regression-kriging method was used for interpolation, in which coded topsoil particle size from the KPP basic samples was included as an explanatory variable (a total of 158,478 georeferenced samples). The sand and clay distribution model was calculated for a 20 × 20 m cell size grid and was adapted to agricultural soils in Slovakia in accord with the database of Bonited Soil and Ecological Units (BSEU). BSEU are classification and identification data expressing the quality and value of the productive-ecological potential of agricultural land in a given habitat expressed by a 7-digit code (referring to climatic region, main soil unit, slope and exposure, soil depth, particle size).

The spatial model of sand, silt and clay content from KPP data was compared with the model from LUCAS data and current mapping in individual KPP samples. In the ArcMap program, a model of particle size change in individual model cadastral territories was created using a map calculator.

Field mapping identified those sites were where there was a clear accumulation of silt particles and thus a significant change in the particle composition of soils. The multiple regression Equation (1) of Fryrear et al. [23] was used to calculate the erodible fraction (EF) of soils based on soil texture and chemical properties [44]:

(1) $$\mathrm{EF}=\frac{29.09+0.31S_{a}+0.17S_{i}+0.33S_{c}-2.59\mathrm{OM}-0.95{\mathrm{CaCO}}_{3}}{100}$$

where S_a_—the soil sand content, S_i_—the soil silt content, S_c_—the ratio of sand to clay contents, OM—the organic matter content and CaCO_3_—the calcium carbonate content. All variables are expressed as a percentage.

Based on the granular composition and chemical properties in the soil, a spatial model of erodible fraction from KPP dataset for interest cadastral areas was created using spatial models at ArcGIS with a map calculator. It was then compared with the European model, which was created by Borrelli et al. [27].

By interpolating the data obtained by our own mapping and particle analysis, we created a spatial model (current state—2016) of sand, silt and clay distribution using the interpolation method “Topo to raster”. The calculated model was then compared with the Balkovič model [75]. As a result we obtained a change in the percentage of individual fractions between years 1961–1970 and 2016.

To analyse occurrence frequency of the analysed variables on the basis of the maps of KPP changes and LUCAS data base, bar graph (histogram) was used. Values on the *x*-axis represent the lower and upper limits of each interval.

In order to verify the results of the change analysis, a study of land cover variability was carried out on the basis of 1990, 2006 and 2018 Corine Land Cover (CLC) maps.

Statistical dependence between KPP, LUCAS and current data using the Spearman Rank Correlation Coefficient was determined, the monotonic relationship between two variables was analysed at the 95% confidence level.

The signed-rank test was used to compare two related, matched samples of soil types in different monitored periods in order to assess whether the mean ranks differ (i.e., a paired difference test). Using the Kruskal-Wallis test, the differences in ratio of soil particles were compared over the monitored periods. Differences in KPP, LUCAS and current data were examined by Bonferroni and LSD range tests [76].

## 3. Results and Discussion

### 3.1. Models of Spatial Distribution of Soil Fractions

**Figure 2:**
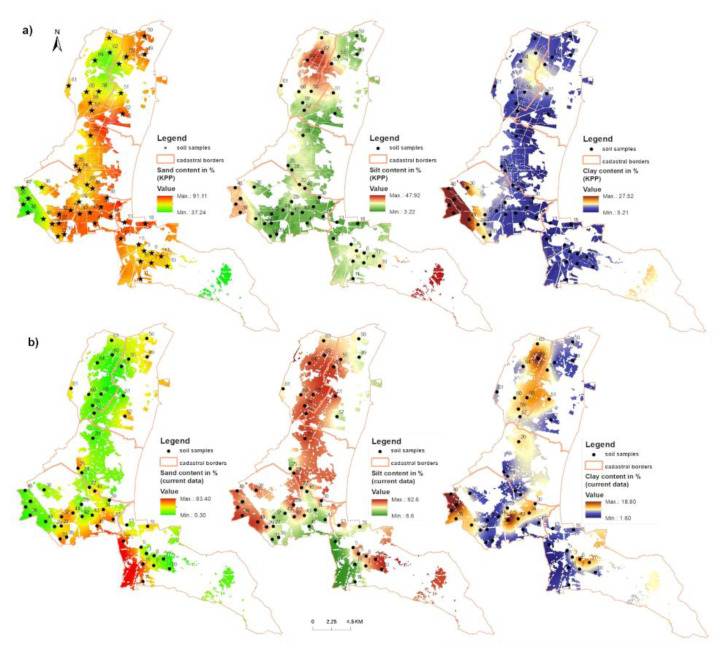
Models of particle size distribution in selected cadastral areas: (**a**) KPP, (**b**) current data (2016).

**Figure 3:**
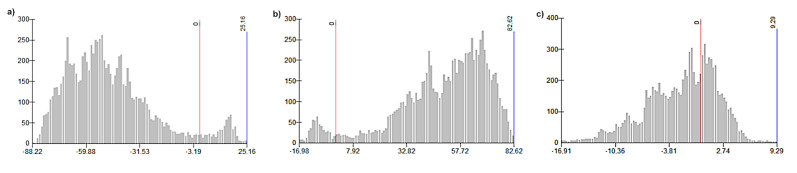
Histograms of percentage change of: (**a**) sand, (**b**) silt, (**c**) clay fractions in years 1961–2016 based on KPP and current data (number of pixels versus percentage change of fraction content).

With the use of GIS tools, models of spatial distribution of sand, silt and clay content were developed based on field measurements (Figure 2). The comparison of spatial models (KPP and current state) of sand, silt and clay distribution shows the ratio of areas (total of 9569 pixels) with minus values decrease in the fraction of sand particles and plus values increase in the amount of fraction of sand particles. The sand fraction change ratio was 8972:597 (Figure 3a). The silt fraction showed the ratio of 493:9076 (Figure 3b). For the clay fraction, the ratio of cells with negative values to positive values was 6147:3422 (Figure 3c).

### 3.2. Changes of Particle Size Distribution

**Figure 4:**
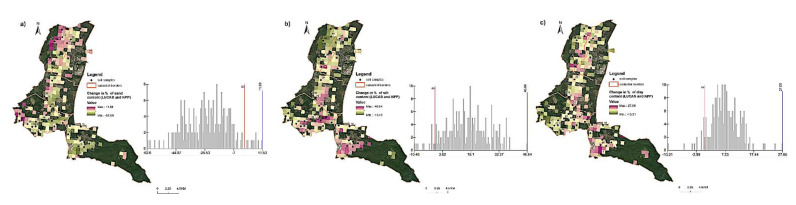
Change in percentage of: (**a**) sand, (**b**) silt, (**c**) clay fractions in 1961–1970 (KPP) and 2009 (LUCAS) in cadastral areas of interest. The histograms present the frequency of occurrence of the values of the analysed fractions.

The spatial model shows that there was a significant decrease in the percentage of sand fraction content in the cadastral areas. The minus values in figure (Figure 4a) mean a decrease and the plus values mean an increase in the percentage of the particle content in the period 1961–2009. The histogram shows that the number of pixels (negative:positive values) for the sand fraction is a 207:9 ratio, for the silt fraction the ratio of 11:205 (Figure 4b), and for the clay fraction the ratio of 14:202 (Figure 4c), and the total number of cells for study area is 216. The results show that in the model area the prevailing erosion type has the character of sedimentation or accumulation due to transporting the finest particles over short distances. The highest transport increment was identified in the silt fraction (0.05–0.002 µm).

**Figure 5:**
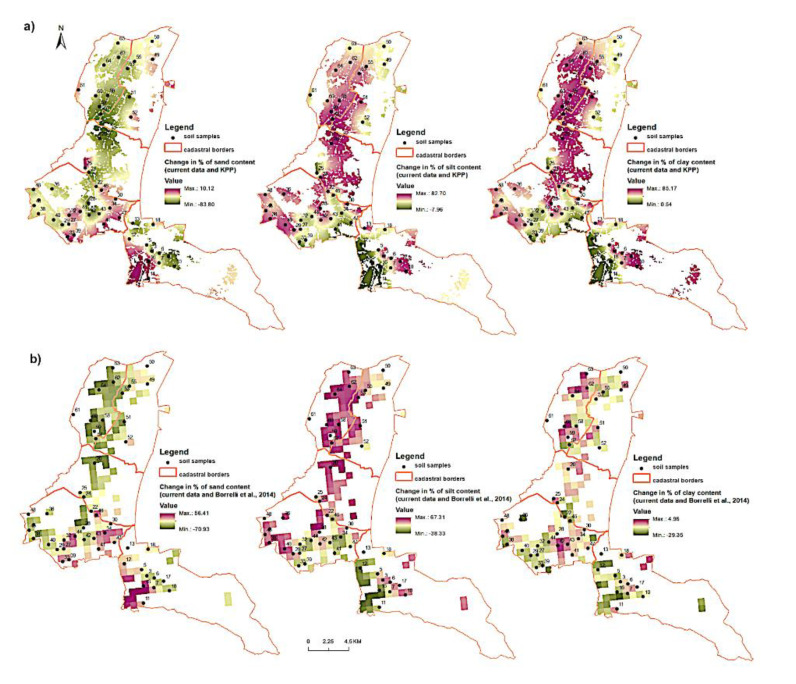
Change in percentage of sand, silt and clay fractions: (**a**) current data and KPP, (**b**) current data and LUCAS.

The comparison of the results of samples from the KPP base and current measurements proved a clear decrease in sand content in the soil of the three northern cadastral units and at the same time an increase in both silt and clay content (Figure 5a). On the maps of changes in the years 2009 and 2016, a significant decrease in sand and a substantial increase in clay content can be observed. In case of clay, no major changes were noted (Figure 5b).

**Figure 6:**
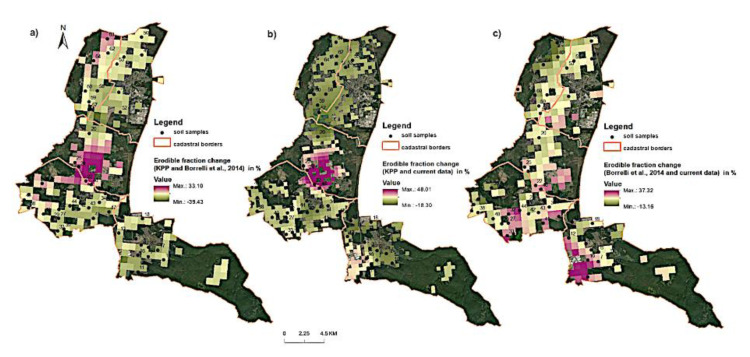
The map of erodible fractions percentage change, calculated based on difference between EF and: (**a**) KPP and LUCAS data, (**b**) KPP and current data, (**c**) LUCAS and current data.

The largest changes in the proportion of erodible fraction were recorded in the middle part of the study area (the cadastral area named Plavecký Štvrtok) with the increase of 33% to 48% (Figure 6). This result is mainly explained by high organic matter (OM) content decrease. The results may be also related to prevailing wind direction (southeast). There is an evident movement of finest soil particles towards southeast direction. Another possible explanation is the depth of aeolian sands which were mainly formed during Pleistocene period as well as OM and CaCO_3_ content change in the study area.

**Figure 7:**
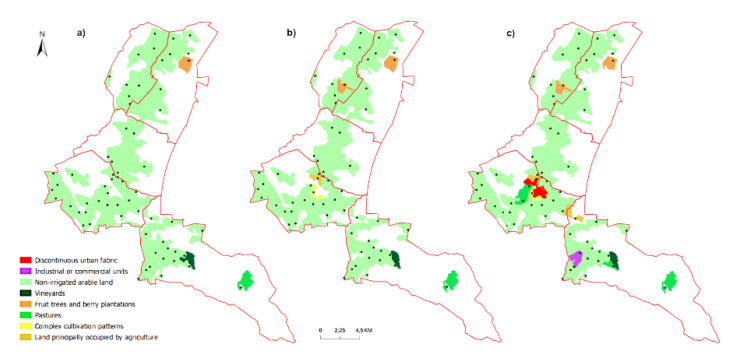
Corine Land Cover maps for the year (**a**) 1990, (**b**) 2006, (**c**) 2018 for the selected georeferenced probes of the study area (Source: Copernicus processed by authors).

On the basis of CLC 1990–2018 maps for selected locations, it was observed that in the central part of the study area (Láb town), there were mainly changes in the terrain cover associated with the transformation of the crop and plot structure and the development of urban areas (Figure 6 and Figure 7). On the other hand, the changes in the Lozorno cadastral unit were the result of the development of industrial areas and soil degradation in this region (Figure 7b,c).

### 3.3. Statistical Analysis of Changes of Soil Properties

**Figure 8:**
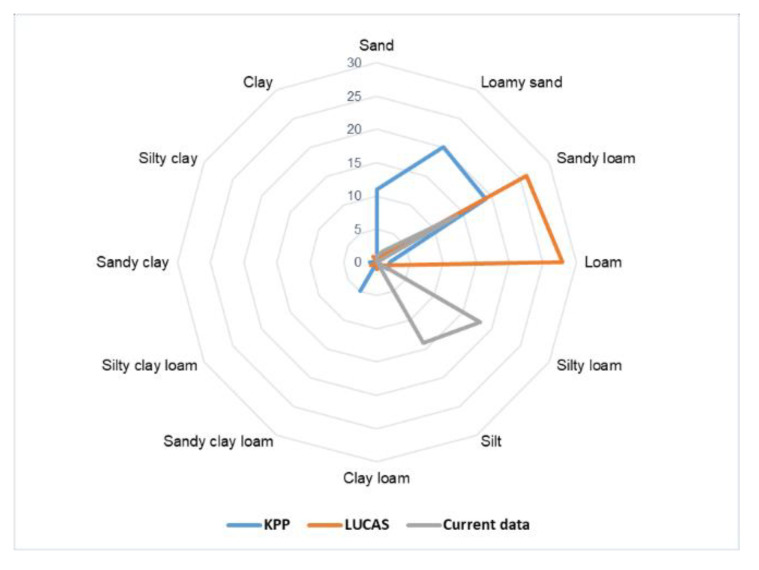
Soil type changes [%] over the years 1961, 2009 and 2016 (KPP, LUCAS and current data). Presentation of a growth trend of soil types with a significant proportion of silt particles (silt, silt-loamy soil).

The particle size analysis showed significant changes in both the particle size composition and the change of soil type on the mapped soil units. The percentage of soil types over the monitored periods KPP, LUCAS and current mapping reflects for sandy soils 18%, 0%, 2%, for loamy sand 33%, 2%, 4%, for sandy loam 32%, 44%, 29% and for loam 3%, 47%, 0%, respectively. The other soil types presented themselves rarely. The analysis of the change in the abundance of soil types (Figure 8) in the monitored time period also indicates a trend in terms of an increase of soils with a significant proportion of silt particles (silt, silt-loamy soil) referring to accumulation zones, and a growth trend of sand particles referring to deflation zones.

Based on data correlation between monitored periods, there was a statistically significant negative correlation between sand and silt content in KPP and LUCAS period at the 95% confidence level (−0.60, *p* = 0), indicating that the proportion of sand was declining and silt was rising (Table 1). The correlation between sand and clay content in these two periods was not proven (0.08, *p* = 0.56). However, when the proportion of silt was rising, the proportion of clay was increasing as well (0.70, *p* = 0). Similarly, there was a statistically significant negative correlation between sand and silt content in LUCAS and current data period at the 95% confidence level (−0.45, *p* = 0), indicating that the proportion of sand was declining while silt was rising. The correlation between sand and clay in these two periods was not proven (0.11, *p* = 0.45). When the proportion of silt was rising, so was the proportion of clay (0.74, *p* = 0). There was a statistically significant negative correlation between sand and silt content in KPP and current data period at the 95% confidence level (−0.66, *p* = 0) indicating that the proportion of sand was declining while silt was rising. The correlation between sand and clay content in those two periods was significantly positive (0.56, *p* = 0). However, there was no correlation between silt and clay (0.19, *p* = 0.18).

From the results achieved in the analyses, it was not possible to evaluate the final trend of change of particle fractions of sandy soils due to wind erosion, but they may be the basis for further possible direction of research of parameters influencing this change (e.g., mapping density, meteorological parameters).

**Figure 9:**
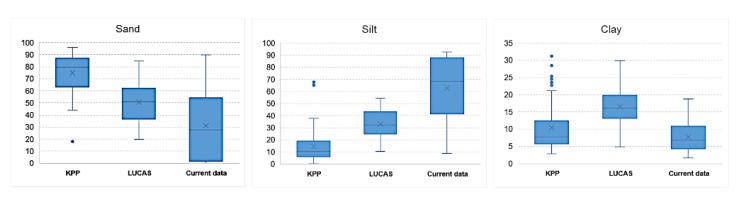
Change of the particle fractions of KPP (1961), LUCAS (2009) and current data (2016).

Comparing the data from KPP (1961), LUCAS (2009) and current data (2016), a significant reduction in the content of sand particles and an increase in the content of silt and clay particles was observed (Figure 9). Box plots refer to average values of sand, silt and clay fraction over three periods. The average of sand content in KPP was about 75%, LUCAS 50%, and current mapping 31% (with high variance). For silt, an increase in KPP of 15%, LUCAS of 33% and current data of 63% was observed, respectively. On the other hand, the average content of clay fractions for particular periods was at a similar level: 10%, 16%, 8%.

The Kruskal-Wallis test verifies the null hypothesis that the medians within each of the three periods are equal. The sand content is significantly different in all monitored periods (Test statistic = 71.59, *p* = 0). The range tests indicated that these three different groups of sand particles were not similar to one another at all. The portion of sand is significantly declining over the monitored period. Similarly, the portion of silt is significantly different in all monitored periods (test statistic = 97.52, *p* = 0). In this case, the range tests indicated three different groups of silt particles not similar to one another. The silt content is significantly rising over the monitored period. The portion of clay is significantly different in monitored period as well (Test statistic = 56.28, *p* = 0), however, range tests indicated difference only between KPP and LUCAS, and LUCAS and current data, but not between KPP and current data.

It may be due to the fact that this is the area where the transport of the finest particles dominates (south-east wind direction) and their accumulation at a short distance from the area where erosion processes take place. A change in the particle size distribution is evident in favour of the fraction of the silt particles.

Wind erosion causes textural changes of topsoil, however the mechanism of particle selection still remains unclear [77,78]. Most studies have confirmed the rule that silts are the particles which are removed in a greater proportion [77,79]. In loamy sand soils silt and clay particles are mostly removed, in sandy loam soils—silts and fine sands. The highest removal of silt was observed in soils with low sand and high silt content, whereas the highest removal of clay was observed in soils with medium sand content [77]. Silt is easier deflated than sand or clay, as it is characterized by small cohesion, moreover it has rather small particle dimensions. Clay particles exhibit larger cohesion and form aggregates too big for the wind to lift easily [79]. As stressed by Goossens and Gross [78], aeolian dynamics of sand and dust is well understood, while for sand-dust mixtures there are wide knowledge gaps. Sand and dust fraction aerodynamic behaviour differs, transportation mode being the distinguishing factor between the two fractions—saltation is characteristic for sand and suspension for dust. The behaviour of mixture is not the sum of the behaviour of the individual components. In their study of a loamy sandy soil in which particle flow behaved as sand-dust mixture, Goossens and Gross proved that in topsoil with a median grain diameter 40–160 μm fine particles were more easily eroded than coarse, while for topsoil with a median grain diameter <40 μm coarsest particles were more easily eroded.

The analysis shows an opposite trend to that observed in the paper by Li et al. (2007), where it was found that the proportion of soil particles with a size of 250–500 μm had increased significantly, but the proportion of particles of 50–125 μm and <50 μm had decreased. Examples of changes in the particle composition of agricultural soils are also given by Lyles and Tatarko [59] who compared changes in particle composition of the top layer of 0.10 m in ten locations in Kansas during the 36-year period between 1948 and 1984. The proportion of the sand fraction increased. The biggest changes were reflected in medium sandy and sandy (coarse-grained) soils. Lyles and Tatarko [59] found that due to wind erosion within the 36 years, the content of sand particles had increased by 6.5% and the content of silt particles by 7.2%. Tatarko [60] argued that changes in the measured properties were related to implemented land management. The 1948 and 1984 samples showed an average increase in sand content of 7.1% and clay 2.1% on the sites while silt content had had an average decrease of 9.2%. The years 1948–1984 were a time of soil degradation in study sites in western Kansas. Subsequent adoption of mitigation measures stabilized, and in some locations reversed, soil degradation, which was proven in research conducted in the years 1996–2011.

In their research, Leys and McTainsh [58] found that wind erosion had caused a particle size increase of >250 µm and a particle diameter of 75–210 µm and <2 µm over a 20-week period. In the wind erosion process, soil particles with a size of 50–125 microns (very fine sand) and <50 μm (silt and clay) were significantly removed during the 2 years of the experimental period. These observations suggest that fine soil particles were preferentially carried away by an increased wind erosion [61]. According to the method of Okin et al. [80] which deals with the calculation of soil nutrient lifetime, the researchers [81] estimated that at 2.15 kg m^2^ per year, silt and clay particles could be carried away within 5 years and all very fine sands (50–125 μm) could be eroded within 10 years.

One of the direct consequences of wind erosion is the loss of soil and the associated loss of soil nutrients by saltation for particles >50 μm and vertical emissions of fine particles. Saltation is primarily responsible for the redistribution of the soil surface in the ecosystem and affects vegetation and soil on a local scale [61,82,83]. The emissions of soil particles with a diameter of <50 μm have a significant impact on the soil nutrient content of surrounding soils, but also on ecosystems, even those several thousand kilometres away [84].

Zobeck and Fryrear [85] studied the physical characteristics of eroded soil in northern Texas and found that soil collected at 0.15 m depth had had visibly different particle composition characteristics compared to samples captured at greater depths. According to research by Gillette [86], the zone of saltation was defined between 0.30–0.45 m depending on the particle size of the soil, wind speed, and physical disturbances.

This research has confirmed that significant changes in the particle composition (e.g., loss of fine particles) caused by erosion can occur relatively quickly, and the finest particles can erode or can be transported over the soil surface within a few years as a direct consequence of wind erosion. Saltation (the flux of sand particles) primarily causes redistribution of the upper soil and affects vegetation and soils on a local scale [87]. Our results show that the surface of the soil in some places has become significantly rougher in over 50 years, but in most areas of interest, the erosive phenomenon was manifested in the form of accumulation of a fraction of silt particles (Figure 3) The analysis of changes in the abundance of soil types in the monitored time period indicates a trend of growth of soil types with a significant proportion of silt particles (silty, silt-loamy soil) (Figure 8).

We also monitored the areas where soil (mainly) silt particles are accumulating or are slowly transported in south-east direction, which corresponds with prevailing winds in this area. Our results have confirmed that the effects of erosive activity are also reflected in the places of impact of eroded particles and in these areas there is a change in the ratio of particle distribution in favour of silt and clay particles. Thus they are called “wind zones”. So far, authors dealing with this issue have only examined areas from which fine particles are carried. However, within our area of interest there are also sites where the accumulation of the finest particles occurs (in the area of windbreaks or forest edges, sloping soil units). The results clearly show the change in particle composition of eroded soils, although no clear trend dependence of the change in time was found.

## 4. Conclusions

Wind erosion is a phenomenon known for years, affecting more than one third of the Earth’s land surface and contributing to soil degradation. Climate change and extreme weather events observed with increasing frequency recently are contributing to a significant intensification and acceleration of erosion processes. Research using spatial data available at an ever-improving resolution is now a cornerstone of soil management. The authors have analysed and discussed the results of a temporal-spatial analysis of the granulometric composition of soils covering more than 50 years. In this paper, historical data from KPP (1961–1070) and from LUCAS data base (2009) have been combined with the authors’ own research results from 2016 with the aim of obtaining more consistent, accurate and long-term information on wind erosion processes in Záhorie Lowlands in Slovakia.

The following results have been obtained:

(1) Methods for integration of data on granulometric composition of studied soils from three different sources using statistical and geostatistical methods have been selected and applicated. The models of spatial distribution of sand, silt and clay content based on long-term archival data and current field measurements for the study site in Záhorie Lowlands have been developed.
(2) A spatio-temporal analysis has shown a significant influence of wind erosion on the granulometric composition of soils in the study area in Slovakia. Soil grains and particles from the layer up to 5 cm depth were transported by the wind, and accumulation of dust fraction dominated in the analysed area. This led to significant changes of soil types, the soil surface become much rougher.
(3) The results obtained from the integrated GIS-based approach can be used in decision support systems and sustainable soil and land management for planning of cost-effective targeted mitigation measures or their evaluation in the process of adaptive management.

## Figures and Tables

**Table 1:** Correlation matrix presenting statistical dependence between the individual variables.

KPP	Sand	1											
	Silt	−0.93	1										
	Clay	−0.71	0.41	1									
	Soil type	−0.72	0.48	0.88	1								
LUCAS	Sand	0.29	−0.28	−0.20	−0.22	1							
	Silt	−0.24	0.26	0.11	0.14	−0.96	1						
	Clay	−0.33	0.25	0.34	0.30	−0.86	0.68	1					
	Soil type	−0.35	0.28	0.34	0.30	−0.68	0.58	0.72	1				
Current data	Sand	0.43	−0.32	−0.44	−0.39	0.20	−0.19	−0.19	−0.18	1			
	Silt	−0.42	0.32	0.41	0.37	−0.19	0.18	0.17	0.16	−0.99	1		
	Clay	−0.33	0.19	0.41	0.34	−0.18	0.14	0.22	0.20	−0.70	0.59	1	
	Soil type	−0.40	0.34	0.34	0.34	−0.16	0.16	0.13	0.15	−0.91	0.92	0.55	1
		Sand	Silt	Clay	Soil type	Sand	Silt	Clay	Soil type	Sand	Silt	Clay	Soil type
		KPP				LUCAS				Current data			

## Data Availability

Sources of the open data are indicated in the text.
